# The role of working memory capacity in soccer tactical decision making at different levels of expertise

**DOI:** 10.1186/s41235-023-00473-2

**Published:** 2023-03-29

**Authors:** Dragan Glavaš, Mario Pandžić, Dražen Domijan

**Affiliations:** 1 Catholic University of Croatia, Zagreb, Croatia; 2 Department of Psychology, Faculty of Humanities and Social Sciences, University of Rijeka, Sveučilišna Avenija 4, 51000 Rijeka, Croatia

**Keywords:** Working memory capacity, Expertise, Deliberate practice, Tactical decision making, Soccer

## Abstract

Athletic skills acquired through deliberate practice are essential for expert sports performance. Some authors even suggest that practice circumvents the limits of working memory capacity (WMC) in skill acquisition. However, this circumvention hypothesis has been challenged recently by the evidence that WMC plays an important role in expert performance in complex domains such as arts and sports. Here, we have used two dynamic soccer tactical tasks to explore the effect of WMC on tactical performance at different levels of expertise. As expected, professional soccer players exhibited better tactical performance than amateur and recreational players. Furthermore, WMC predicted faster and more accurate tactical decisions in the task under auditory distraction and faster tactical decisions in the task without distraction. Importantly, lack of expertise × WMC interaction suggests that the WMC effect exists at all levels of expertise. Our results speak against the circumvention hypothesis and support a model of independent contributions of WMC and deliberate practice on expert performance in sports.

**Supplementary Information:**

The online version contains supplementary material available at 10.1186/s41235-023-00473-2.

## Significance statement

What are the sources of expertise is one of the central topics in cognitive psychology. In this regard, the important question that remains unresolved is whether working memory capacity contributes to experts’ performance or its limits could be circumvented by many hours of deliberate practice in a specific domain. Here, we examined this question in the context of decision-making in sports. We asked soccer players at different levels of expertise (professional, amateur, and recreational) to judge the next best move for a player shown in a short video clip. The video clips depicted real-life situations routinely encountered by players during a match. The results provide clear evidence for a unique contribution of working memory capacity to the decision-making performance at all levels of expertise. Such findings support models that assume the existence of a general or domain-free control mechanism with limited capacity whose impact on behavior cannot be overridden by extensive practice in the domain. In other words, our data suggest that working memory capacity and deliberate practice make independent contributions to expert performance in sports.

## Introduction

The view that expert performance is largely, and perhaps even entirely, a reflection of training history has held sway in the scientific literature on expertise for decades (Ericsson et al., 1993). This view was championed by Ericsson and colleagues (Ericsson et al., 1993), who argued that innate "talent", genetically prescribed traits, and characteristics, play little if any direct role in expert performance, except in the case of body size and height (Ericsson et al., 2007). This "nurture" view of expert performance is also popular among non-scientists. For example, reflecting on his career, the former basketball superstar Michael Jordan once commented, "I practice as if I am playing the game. So, when the moment comes in the game, it is not new to me. That is the beauty of the game of basketball; that is the reason why you practice; that is the effort. So, when you get to that moment, you do not have to think. Instinctively things happen."

A testable prediction that follows from Ericsson and colleagues’ theory has been termed the "circumvention-of-limits hypothesis" (Hambrick & Meinz, 2011). According to this hypothesis, through extended deliberate practice "performers can acquire skills that circumvent basic limits of working memory capacity (WMC) and sequential processing" (Ericsson & Charness, 1994, p. 725). In particular, deliberate practice leads to the development of extended or long-term working memory (LT-WM) that enables experts to bypass reliance on WMC in the performance of domain-relevant tasks.

LT-WM is a domain-specific portion of long-term memory which relies on efficient encoding strategies and retrieval structures that facilitate memory storage and retrieval (Ericsson & Kintsch, 1995). It is thought that LT-WM contributes to the superior performance of expert chess players, as revealed by seminal studies on expertise (Chase & Simon, 1973a, 1973b; de Groot, 1965) and to the superior perceptual-cognitive skills exhibited by experts in many sports (Mann et al., 2007; Williams & Ford, 2008).

The *circumvention-of-limits hypothesis* received support from studies showing diminished or no effect of WMC on expert performance in domain-specific tasks. For example, in the study of Hambrick et al. (2012), visuospatial ability predicted geological bedrock mapping performance at low but not at high levels of geological knowledge. Furthermore, Sohn and Doane (2003) found the LT-WM × WMC interaction revealing a reduced impact of WMC on aviation-situation awareness among more skilled pilots.

However, there is also evidence that individual differences in many domains are not just a product of deliberate practice but also depend on cognitive abilities (Hambrick & Meinz, 2011; Hambrick et al., 2014a, 2014b; Macnamara et al., 2014, 2016). Hambrick et al. (2016) emphasized that WMC, or the capacity to control and coordinate processes and storage during the performance of complex cognitive tasks (Miyake & Shah, 1999), is a significant piece of the expertise puzzle because it regulates and maintains relevant information in an active state in the service of complex cognition. Support for this *independent influence hypothesis* (e.g., Hambrick & Oswald, 2005) comes from studies showing that WMC affects performance even in skilled artists and athletes. Meinz and Hambrick (2010) found that the positive effect of WMC on piano sight-reading exists in pianists with high as well as with low levels of deliberate practice. The additive effect of WMC, independent of domain-specific knowledge, has also found empirical support in baseball-related tasks. WMC positively predicted performance beyond and independent of knowledge in the baseball and baseball-analogy spaceship task (Hambrick & Oswald, 2005) and in tracking baseball game-relevant and irrelevant information (Hambrick & Engle, 2002). Furthermore, Meinz et al. (2012) found WMC to be an equally important predictor of performance on crucial poker skills at low and high knowledge levels of Texas Hold'Em poker. These findings challenged the circumvention-of-limits hypothesis and became the subject matter of the intense scientific debate on the relative contributions of deliberate practice and WMC on expert performance (Ericsson, 2014, 2016; Hambrick et al., 2014a, 2014b).

Contrasting findings regarding the role of WMC in expert performance suggest that there may exist a potential moderator variable that conceals the effect of WMC in some studies. Hambrick et al. (2012) proposed that a type of task used in the study is such a moderator variable. Unchanging input in static tasks, such as bedrock mapping (Hambrick et al., 2012), allows skilled participants to employ efficient encoding strategies and robust retrieval structures. In this way, they circumvent the need to engage capacity-limited working memory. By contrast, continuously changing input in dynamic tasks make it difficult for the participant to rapidly encode information and retrieve it from long-term memory using knowledge-based retrieval structures. Consequently, dynamic tasks engage working memory to a greater degree, which increases the chance of detecting the WMC effect even in highly skilled performers. Furthermore, dynamic tasks have a higher ecological validity because they incorporate some of the critical components of different sports, which are, according to Moran (2009), a "rich and dynamic laboratory for the study of how the mind works" (p. 422).

Furley and Memmert (2010) argued that the field of team sports is a promising avenue for testing and advancing cognitive psychological theories. Along these lines, Furley and Memmert (2012) were the first to employ dynamic tactical decision-making tasks to study the role of WMC in the tactical performance of team-ball sports. In two experiments, the authors investigated whether WMC is predictive of tactical performance under distraction and additional demand to resolve response competition. In Experiment 1, the authors utilized a time-constrained tactical decision-making task under auditory distraction with stimuli consisting of basketball game stills representing offensive tactical decisions. Participants had to decide whether a marked player with a ball should shoot, cut/dribble or pass the ball within 1,000 ms of stimulus presentation and 750 ms of the fixation cross. Distraction stimuli, auditory information that should be ignored during tactical decision-making task, was modelled on the selective attention paradigm and dichotic listening studies (Conway et al., 2001; Wood & Cowan, 1995). High-WMC athletes were expected to exhibit better tactical performance while inhibiting distraction and focusing on tactical tasks.

In Experiment 2, participants decided whether the player holding the puck should shoot, pass, or make a solo effort within 1,000 ms of stimulus presentation and 3,000 ms of the mask. Specific to Experiment 2, along with regular trials, the ice hockey decision-making tasks contained team time-out trials, in which the recommended tactical decision for the following situation was valid 66% of the time. However, in the rest of the time-out trials, recommended tactical decision was not optimal. By inducing interference conditions as in the Stroop paradigm (Kane & Engle, 2003; Long & Prat, 2002), authors hypothesized that high-WMC athletes would be less likely to follow non-valid tactical recommendations in time-out trials and better adjust tactical decisions to the current situation.

The results showed that higher WMC predicts higher accuracy in tactical decisions of basketball players under distraction and hockey players in interference conditions. Furthermore, high-WMC basketball players were better at focusing on tactical decisions, as they detected their name in the distracting auditory message less frequently than low-WMC basketball players. This study provided unique and valuable evidence of the role of WMC in ball-sports situations.

Despite its novelty and theoretical relevance, several aspects of Furley and Memmert's (2012) study raise a concern. For example, they used an extreme-group design, whereby only participants who achieve highest and lowest scores on the relevant variable are taken into analysis. In the present context, this involves creating a set of discrete categories from a continuous WMC measure and analysing only those categories representing the upper and lower end of the WMC distribution. Commonly, scores that fell in the upper and lower quartile are used in the analysis (Conway et al., 2005). Given the very pointed distribution of WMC scores in their study, in contrast to the distribution that Kane et al. (2004) observed in the same task, Furley and Memmert (2012) classified the highest 20% of the WMC distribution as high-WMC athletes and the lowest 20% as low-WMC athletes. The discretization of a continuous variable discards variation in individual scores and may result in increased Type I error, spurious correlation, and inefficient, distorted or less accurate effect size estimates (Conway et al., 2005; Gelman & Park, 2009; Iacobucci et al., 2015). This concern is even more exaggerated with the small number of participants in the study of Furley and Memmert (2012) (*n* = 28). In addition, the sample in their study did not include professional or expert-level athletes. Thus, it was not possible to conclusively answer whether expertise moderates the impact of WMC on tactical decision-making.

A recent study by Vaughan and Laborde (2021) on basketball players at different levels of expertise (including elite and super-elite athletes) examined the moderating role of expertise in the relationship between spatial working memory and free-throw performance. The results showed a slightly stronger relationship between visuospatial WMC and free-throw performance among super-elite and elite compared to amateur and novice youth basketball players, suggesting a moderating role of athletic expertise. This study, together with another study showing a correlation between the visuospatial ability of young soccer players and several measures of soccer performance (Glavaš, 2020), supports the theoretical relevance of the working memory capacity in sports. However, both studies tested only the visuospatial component of the working memory (Baddeley, 2003) because they used the Corsi-Block task (Corsi, 1973) to assess the visuospatial short-term memory span. Furthermore, Glavaš (2020) did not operationalize skill levels at all, whereas the average age of the sample in Vaughan and Laborde's (2021) study was slightly above 19 (*SD* = 1,01). This raises the question of the exact level of expertise of these athletes. More specifically, although Vaughan and Laborde (2021) classified elite and super-elite athletes based on Swann et al.'s (2015) recommendations, their participants were primarily involved in training and competition (3,3–9,7 years) below-senior levels. Thus, although participants were engaged in deliberate practice, they still might not reach truly expert levels of performance that would be less dependent on basic cognitive abilities. Finally, Vaughan and Laborde (2021) measured performance using only a single task, a basketball free-throw task.

To address these shortcomings and provide a more rigorous test of the circumvention-of-limit hypothesis, we compared the speed (reaction time) and accuracy of tactical decision-making among three groups of participants (professional, amateur, and recreational senior soccer players) representing different levels of expertise and tested whether WMC distinctively contributes to the tactical performance of skilled and less skilled athletes. To assess the WMC, we administered two working memory span tasks that assess capacity for maintenance and executive control as functions of the entire working memory system. Furthermore, to remove task-specific factors, we derived the latent variable from the common variance of the results of two tasks (Conway et al., 2005).

We developed the tactical decision-making tasks following Furley and Memmert's (2012) study, except that we used video clips from real games to increase ecological validity. We also manipulated the presence of auditory distraction during decision-making. To test the main and interaction effects of WMC and expertise, we utilized moderation analysis with the multi-categorical moderator.

## Method

### Participants

A total of 129 male adults, professional (*N* = 42), amateur (*N* = 46) and recreational (*N* = 41) soccer athletes, took part in the study. Professional soccer players (*M*_age_ = 26.36, *SD* = 5.58) were members of the Croatian First League and Bosnia and Herzegovina Premier League clubs. On average, they have been competing at the highest national level for 6.71 years (*SD* = 4.37, *Range* = 2–17). Around two-thirds of professionals (64.3%) entered a senior professional level at the age of 18. The rest of the professionals started competing on a professional level by the age of 22 at the latest. Most of them (71.4%) started soccer training at the age of seven (the rest at the age of eight) in elite national soccer schools or academies. No athlete reported a training hiatus other than injuries not longer than three months (2 players). All professional athletes reported mentored individual soccer-specific training in addition to the structured training in a club during seasons and off-seasons. The amateur soccer players (*M*_age_ = 24.61, *SD* = 5.17) have been competing in the third (43.5%) and fourth (56.5%) national competitive levels of Croatia and Bosnia and Herzegovina for 4.78 years (*SD* = 3.37, *Range* = 1–15) on average. The majority of amateur soccer players (92.8%) had begun soccer training between ages 7 and 11 in lower-league soccer schools. Recreational soccer players played soccer or futsal for recreation, mostly once (34.1%), twice (36.6%), or three times (29.3%) a week. We excluded two participants from the dataset because one amateur player failed to complete the tactical decision-making task under distraction, and one professional player failed to complete the Operation Span task.

Given that Meinz and Hambrick (2010) did not detect the assumed medium-sized interaction effect between deliberate practice and WMC, we predicted a minor reduction of the impact of WMC on tactical decision-making at a professional level of soccer expertise. Thus, power analysis indicated that 124 participants would be adequate to detect the small to medium-sized interaction effect (Cohen's *f* ^2^ = 0.08) between expertise and WMC.

### Apparatus

Participants were tested individually in a session that took approximately 40 min to complete. Participants were seated approximately 60 cm from a PC monitor (17-inch screen). The stimulus presentation and data collection were controlled by the E-prime Professional (Version 2.0; Schneider et al., 2002) software package for running psychological experiments.

### Materials and procedure

First, we interviewed participants about their expertise and soccer experience. Following interviews, participants completed the tactical decision-making task, first without distraction and then under auditory distraction. Half of the participants then took the Operation Span, followed by Symmetry Span, whereas the other half completed tasks in reverse order.

#### Level of expertise and soccer experience interview

We interviewed participants regarding their soccer training history, including the age of training onset, the leagues, clubs they played in, and any soccer training outside of the structured club training. The interview included detailed questions about soccer practice, competitive levels, experiences from the first years of training, and other activities such as recreational playing, watching soccer games, and playing video games (e.g., FIFA, PES).^1^ Professional soccer players were categorized as experts if they were involved in structured soccer training in clubs or academies for ten or more years and played professionally for not less than two seasons. Players were categorized as amateurs if they played for third and fourth national league clubs. Before the senior level, they trained in lower-level league clubs. The recreational soccer players were involved in structured training, played competitively for no more than three years, and played soccer or futsal for recreational purposes afterwards.

#### Tactical decision-making tasks

The tactical decision-making task and tactical decision-making task under distraction consist of 25 soccer video clips each. From a pool of 122 soccer video clips taken from professional league matches, two soccer experts (UEFA A and UEFA B license) chose the offensive game situations with the highest consensus on the correct tactical decision in that situation (in their expert opinion). Another two soccer experts (UEFA Pro and UEFA A license) independently verified these decisions by offering their opinion on the correct tactical decisions in that subset of video clips. In the final stimulus set, we included only those clips where all four soccer experts agreed on the best tactical decision to be made by a player.^2^

To familiarize themselves with the procedure, participants completed the block of practice trials, including 12 tactical video clips not used in the main study.

In the tactical video clips, an athlete with the ball was marked with a red arrow, while two tactical options (either two passes or a pass and a shot on goal) were marked with numbers 1 and 2 in yellow. Participants had to make tactical decisions by pressing the corresponding number on the keyboard (1 or 2). The duration of video clips varied from 2000 to 4500 ms. The last frame of each video clip remained on the screen for 1,000 ms, followed by a white screen (1,500 ms), during which responses were collected.

**Fig. 1:**
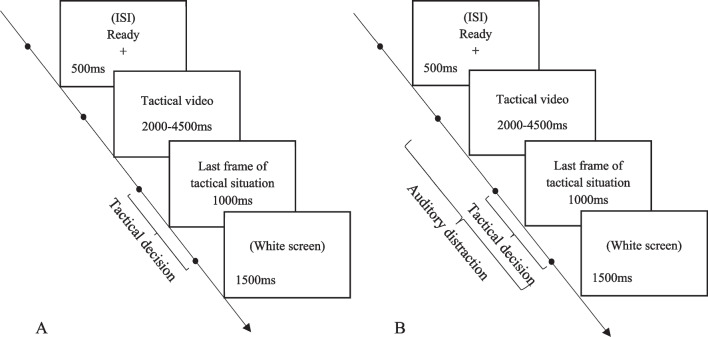
Illustration of a trial sequence from (**A**) tactical decision-making task without distraction and **B** tactical decision-making task under auditory distraction. *Note.* ISI = interstimulus interval

In the tactical decision-making task under distraction, we adopted a dichotic listening task, developed by Cherry (1953) to study auditory selective attention. In the dichotic listening task, participants are instructed to listen and repeat unrelated words presented through a pair of headphones to one ear (attended channel) and ignore another set of unrelated words presented to the other ear (unattended or ignored channel). Using this procedure, Moray (1959), Wood and Cowan (1995) and Conway et al. (2001) studied the "cocktail party effect", which refers to the finding that a highly salient word, such as a participant’s own name, sometimes captures attention when presented in the ignored channel. For example, Wood and Cowan (1995) found that only 34.6% of participants noticed their name in the ignored channel, and Conway et al. (2001) found that the low-WMC participants detected their own names more often. Conway et al. (2001) suggested that low-WMC participants were less able to inhibit a distracting environment and less successful in focusing their attention on the primary task, which consequently led to higher detection of their names in the ignored channel. Furley and Memmert (2012) adopted this paradigm to tactical decision-making in sports so that tactical decision-making was the primary task while the auditory stream was distracting stimulus. A distracting auditory stream contained mono- and disyllabic words recorded in two different monotone female voices (which altered after 56 of 116 tactical stills in total) at a rate of 80 words per minute. The distracting stimuli started simultaneously with the tactical decision-making task and continued during the entire tactical task. The presentation order of the words was identical across all participants. The given name of every participant was digitally inserted 250 ms after the onset of the 103rd tactical still. We adopted this procedure in our tactical decision-making task. We recorded an auditory stream in a monotone male voice at the rate of 80 mono- and disyllabic words per minute and presented them concurrently with the tactical decision-making task. The order of the words in distracting stimuli was identical across all participants. The first name was known for 106 participants due to earlier acceptance of the invitation to participate in the study and was inserted 500 ms before the tactical video still of the 22nd video clip. The neutral word was presented to the rest of the participants (*N* = 23). Participants were instructed to ignore the auditory stimuli and to focus their attention on a tactical decision-making task. After participants completed the tactical task under distraction, they were asked the following questions: (a) "Did you notice anything unusual about the distracting message?", "If yes, what?" (b) "Did you notice your first name in the irrelevant message?". An example of a trial sequence is depicted in Fig. 1.

### Tactical decision-making response times (RTs) and accuracy

To detect outliers for the distributions of decision-making response times (RT) of every participant, we applied the outlier labelling rule (Hoaglin & Iglewicz, 1987; Hoaglin et al., 1986). The rule calculates the interval by multiplying the difference between the third and the first quartile with the coefficient *g* = 2.2 and then subtracting or adding the multiplication result to the first and the third quartile value, respectively. Responses outside the interval were classified as outliers, not resulting from tactical knowledge but as artefacts of other factors. If detected, no more than one (69,09%) or two (30,91%) data points per participant were characterized as outliers. We calculated the central tendency of tactical decision-making speed as the average RT medians of each participant and accuracy as the average ratio of each participant's correct answers.

#### WMC

To measure WMC, we used two computer-administered complex span tasks, a shortened version of the Operation Span (*OSpan*) as a verbal and a shortened version of Symmetry Span (*SymSpan*) as a spatial task (Foster et al., 2015). The OSpan required participants to remember letters while confirming whether the displayed number is the correct answer to a math equation by clicking *True* or *False* (Unsworth et al., 2005). After each series of equation-letter pairs, randomly varied from three to seven, participants selected the letters in the order presented in the 4 × 3 letter grid. The number of letters recalled in the correct order was the total score (the partial score). The maximum score was 25. The SymSpan required participants to remember a red square's location within the 4 × 4 grid while judging whether a displayed figure is symmetrical along its vertical axis by clicking *Yes* or *No* (Unsworth et al., 2009). Symmetry-location pairs randomly varied from two to five, and after each series of pairs, participants recalled red square locations in the order presented. The number of red square locations recalled in the correct order was the total score (the partial score). The maximum score was 14. Prior to the start of the main experiment, participants completed three blocks of practice trials including WMC tasks, in the following order: the storage practice block (i.e., recall of the letters, recall of the square locations), the processing practice block (i.e., math operations, symmetry judgment), and the interleaved practice block analogous to the actual trials.

## Results

To test the effect of WMC on tactical decision-making and its dependence on the level of soccer expertise, we utilized a moderation analysis with a multi-categorical moderator. Given the hypothesis on the superior tactical decision-making of professional soccer players, we used Helmert coding for levels of expertise (Hayes & Montoya, 2017). The regression coefficients (*b*s) of this method estimate the difference between the mean of professional soccer players and the unweighted mean of amateur and recreational soccer players (D_1_) and the difference between the mean of amateur and recreational soccer players (D_2_). The coefficient for WMC (X) is the unweighted averaged conditional effect of WMC on tactical decision-making across the three groups of soccer players. Two interactions effects are the estimated difference of the WMC effect on the tactical decision-making of professional soccer players in comparison to the tactical decision-making of soccer players from two lower levels (D_1_ × WMC) and the estimated difference of the WMC effect on tactical decision-making of amateur in comparison to recreational soccer players (D_2_ × WMC). The model assumes the conditional effect of WMC across the three groups of soccer players. In the Supplemental material (Additional file 1: Table S1), we also provided a correlation matrix showing the correlation between WMC and tactical decision-making across three levels of soccer expertise.

We further examined the (non)existence of expertise × WMC interaction by computing Bayes factors to establish whether a non-significant result supports a null hypothesis over a theory or whether the data are just insensitive (Dienes, 2014; Dienes & McLatchie, 2018). Bayes factors were calculated using the Bayes factor package in R (Morey & Rouder, 2019).

We performed a logistic regression analysis to test the contribution of WMC and expertise and WMC x expertise interaction to the frequency of the own name detection.

We screened data on Ospan, SymSpan, and underlying WMC factor for values higher than 3.5 standard deviations from the sample means (outliers) and found no values higher than the absolute value of *z* = ǀ3.1ǀ (value for OSpan). As expected, the two complex span tasks correlated moderately, *r* = 0.49. As a total WMC score, we specified the unique latent factor underlying the two complex span tasks by calculating the factor scores. We found no differences between recreational, amateur, and professional soccer players regarding Ospan, *F*(2,124) = 2.62, *p* = 0.077, SymSpan, *F*(2,124) = 1.43, *p* = 0.244, and the underlying WMC factor, *F*(2,124) = 2.23, *p* = 0.112. In addition, we found no reaction time-accuracy tradeoff as the correlations between response time and accuracy in tactical decision-making task and tactical decision-making task under distraction were *r* = 0.02, *p* = 0.853 and *r* = 0.01,* p* = 0.894, respectively. Descriptive statistics for complex span tasks scores and the tactical decision-making tasks response time and accuracy are displayed in Table 1.

**Table 1:** Descriptive statistics on the complex span and tactical tasks

		M	SD	Range
Operation Span		17.08	4.52	4–25
Symmetry Span		9.21	3.00	5–25
Tactical decision-making task	RT	669.63	250.54	152–1422
	ACC	0.86	0.08	0.60–1.00
Tactical decision-making task under distraction	RT	669.91	250.45	185–1442
	ACC	0.76	0.11	0.52–0.96

*RT* response time, *ACC* accuracy rates

### Tactical decision making

#### RTs analysis

The results of the moderation analysis showed that the model accounted for 15.5% of the tactical decision-making speed variance, *F*(5, 121) = 4.45, *p* < 0.001. Professional soccer players made faster tactical decisions (*M* = 595 ms, *SD* = 277.06) in comparison to amateur and recreational soccer players (*M* = 705 ms, *SD* = 230.08),^3^*t*(125) = 2.89, *p* = 0.005, whereas amateur soccer players (*M* = 658 ms, *SD* = 212.01) made faster tactical decisions in comparison to recreational soccer players (*M* = 757 ms, *SD* = 240.53), *t*(125) = 2.42, *p* = 0.017. Higher levels of WMC were associated with faster tactical decisions, *t*(121) = − 3.40, *p* < 0.001.

**Fig. 2:**
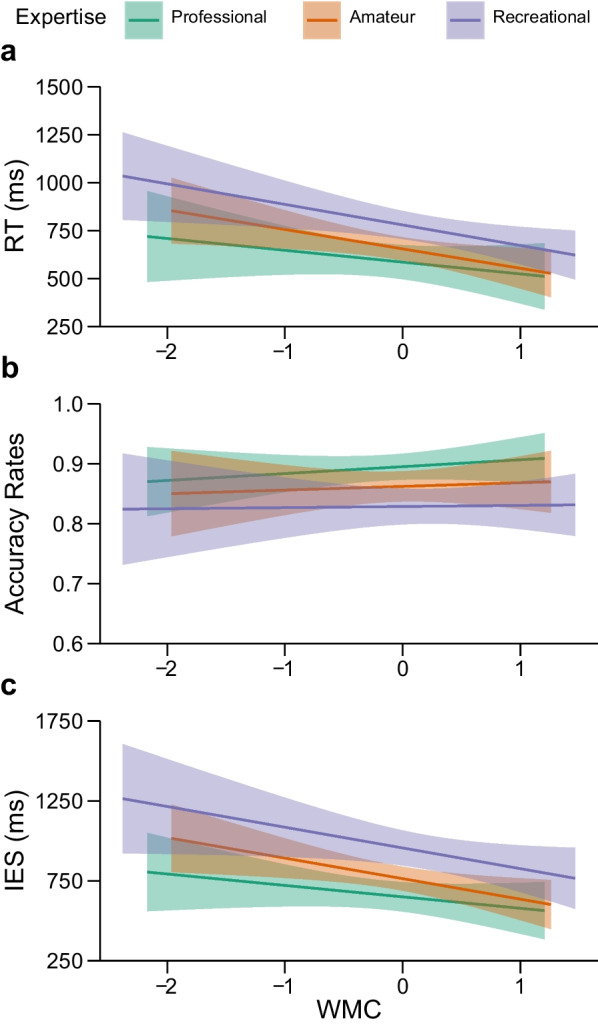
The effect of WMC on the **a** RTs (in ms), **b** accuracy rates, and **c** IESs (in ms) in tactical decision-making at different levels of soccer expertise

The expertise × WMC interactions were not significant, as shown by the comparison between professional soccer players and players with lower levels of expertise (D_1_ × WMC), *t*(121) = − 0.76, *p* > 0.250, and by the comparison between amateur and recreational soccer players (D_2_ × WMC), *t*(121) = − 0.09, *p* = 0.250 (Table 2). The regression slopes are depicted in Fig. 2A.

**Table 2:** Regression (moderation) analysis predicting RTs, accuracy rates, and IESs of tactical decision-making (Helmert coding)

	*b*	*se*	*t*	*p*	95% CI	
					*LL*	*UL*
RT						
WMC	− 90.018	26.490	− 3.40	< .001	− 142.462	− 37.575
D_1_	131.467	45.437	2.89	.005	41.512	221.422
D_2_	124.734	51.543	2.42	.017	22.690	226.777
D_1_ × WMC	− 42.602	56.230	− 0.76	.451	− 154.042	68.838
D_2_ × WMC	− 5.893	64.776	0.09	.928	− 134.133	122.348
Accuracy						
WMC	0.007	0.009	0.71	.481	− .012	.025
D_1_	− 0.050	0.016	− 3.15	.002	− .081	− .019
D_2_	− 0.034	0.018	− 1.88	.063	− .069	.002
D_1_ × WMC	− 0.008	0.019	− 0.38	.701	− .046	.031
D_2_ × WMC	− 0.004	0.022	− 0.19	.850	− .049	.040
IES						
WMC	− 109.890	32.859	− 3.344	.001	− 174.944	− 44.837
D_1_	209.923	56.362	3.725	< .001	98.340	321.507
D_2_	192.070	63.936	3.004	.003	65.492	318.648
D_1_ × WMC	− 57.522	69.824	− 0.824	.412	− 195.756	80.713
D_2_ × WMC	− 1.217	80.350	− 0.015	.988	− 160.292	157.857

D_1_ = professional vs. lower levels of expertise; D_2_ = amateur vs. recreational level of expertise

Because of its theoretical relevance, we further examined expertise × WMC interaction by computing Bayes factors. We fitted the full model with expertise × WMC interaction and compared it to the model without interaction (with the main effects of expertise and WMC included). The results showed that the model without interaction is 11.75 as likely as the model with interaction, given the observed data. This is strong evidence against the expertise × WMC interaction.

#### Accuracy rates

The results of moderation analysis showed that the model accounted for 10.0% of the tactical decision-making accuracy variance, *F*(5, 121) = 2.68, *p* = 0.025. The moderation analysis revealed higher accuracy of professional (*M* = 0.893, *SD* = 0.067) in comparison to amateur and recreational soccer players (*M* = 0.846, *SD* = 0.088), *t*(125) = − 3.15, *p* = 0.002. Slightly higher accuracy of amateur (*M* = 0.862, *SD* = 0.083) in comparison to recreational soccer players (*M* = 0.829, *SD* = 0.090) was at the border of significance, *t*(125) = − 1.88,* p* = 0.063. WMC did not affect tactical decision-making accuracy, *t*(121) = 0.71, *p* > 0.250. In addition, there were no significant expertise × WMC interactions as shown in the comparison between professional soccer players and players with lower levels of expertise (D_1_ × WMC),* t*(121) = − 0.39, *p* > 0.250, and in the comparison between amateur and recreational soccer players (D_2_ × WMC), *t*(121) = − 0.19, *p* > 0.250 (Table 2, Fig. 2B).

As in the RT analysis, we computed Bayes factors to check whether data supports the model without interaction, the model with interaction, or they are just insensitive. The results showed that the model without interaction is 9.64 as likely as the model with interaction, given the observed data. This corroborates the RT analysis by suggesting substantial evidence against expertise × WMC interaction.

#### Inverse efficiency scores (IES)

We also analyzed a combined speed-accuracy measure known as an IES. It is computed by dividing the average correct RT by the proportion of correct responses per participant and per condition. IES can be interpreted as an RT measure corrected for the proportion of errors committed. Analysis on IESs showed that the model accounted for 19.6% of the variance, *F*(5, 121) = 5.92, *p* < 0.001. As in the RT analysis, professional soccer players were faster (*M* = 661 ms, *SD* = 287.97) than amateur and recreational soccer players (*M* = 845 ms, *SD* = 316.85), *t*(125) = 3.73, *p* < 0.001, whereas amateur soccer players (*M* = 769 ms, *SD* = 263.10) made faster decisions than recreational soccer players (*M* = 929 ms, *SD* = 351.31), *t*(125) = 3.00, *p* = 0.003.

Higher levels of WMC were associated with faster tactical decisions, *t*(121) =  − 3.34, *p* = 0.001. The expertise × WMC interactions were not significant, as shown by the comparison between professional soccer players and players with lower levels of expertise (D_1_ × WMC), *t*(121) =  − 0.82, *p* > 0.250, and by the comparison between amateur and recreational soccer players (D_2_ × WMC), *t*(121) = − 0.02, *p* > 0.250. The regression slopes are depicted in Fig. 2C. Bayes factors on IES showed that the model without interaction is 10.65 as likely as the model with interaction, given the observed data. This provides further evidence against expertise × WMC interaction.

### Tactical decision making under distraction

We followed the same procedures as in the analysis of tactical decision-making task without distraction. We used the same data exclusion criteria and fitted the same model to the RTs and accuracy rates with WMC, expertise, and their interaction as predictors. We also used the same Helmert contrasts for the levels of expertise. In addition, we computed Bayes factors to decide on the existence or non-existence of theoretically relevant interaction between WMC and expertise.

#### RTs analysis

**Fig. 3:**
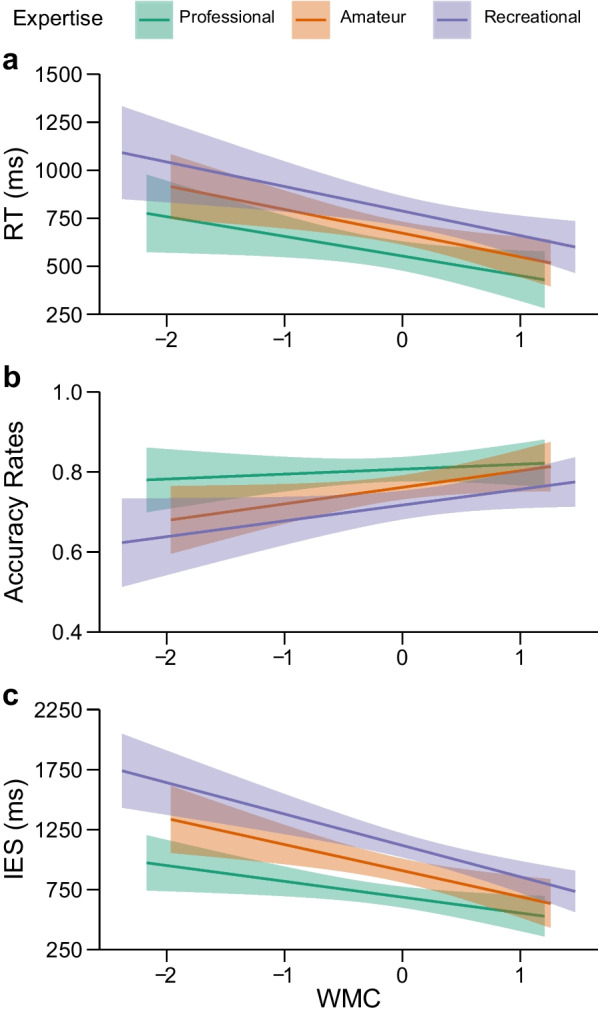
The effect of WMC on the **a** RTs (in ms), **b** accuracy rates, and **c** IESs (in ms) in tactical decision-making under distraction at different levels of soccer expertise

The results of moderation analysis on RTs showed that the model accounted for 23.7% of the tactical decision-making speed under distraction variance, *F*(5, 121) = 7.52, *p* < 0.001. There was a significant effect of expertise showing faster tactical decision-making of professionals (*M* = 570 ms, *SD* = 245.97) compared to lower levels of expertise (*M* = 718 ms, *SD* = 239.51),* t*(125) = 4.09, *p* < 0.001, and faster tactical decision-making of amateur (*M* = 678 ms, *SD* = 214.97) compared to recreational football players (*M* = 761 ms, *SD* = 259.42), *t*(125) = 2.35, *p* = 0.020. There was also a significant effect of WMC showing faster tactical decisions of players with higher WMC, *t*(121) = -− 4.68, *p* < 0.001. There were no significant expertise × WMC interactions across both comparisons: professional vs. amateur and recreational levels of expertise (D_1_ × WMC), *t*(121) = − 0.43, *p* > 0.250 and amateur vs. recreational levels (D_2_ × WMC), *t*(121) =  − 0.07, *p* > 0.250 (Table 3). Regression slopes are depicted in Fig. 3A.

**Table 3:** Regression (moderation) analysis predicting RTs, accuracy rates, and IESs of tactical decision-making under distraction (Helmert coding)

	*b*	*SE*	*t*	*p*	95% CI	
					*LL*	*UL*
RT						
WMC	− 117.848	25.164	− 4.68	< .001	− 167.667	− 68.029
D_1_	176.662	43.163	4.09	< .001	91.210	262.115
D_2_	115.234	48.963	2.35	.020	18.298	212.170
D_1_ × WMC	− 21.108	53.472	− 0.43	.666	− 128.971	82.755
D_2_ × WMC	− 4.376	61.534	− 0.07	.943	− 126.198	117.446
Accuracy						
WMC	0.031	0.011	2.73	.007	.009	.054
D_1_	− 0.068	0.020	− 3.47	< .001	− .106	− .029
D_2_	− 0.044	0.022	− 2.01	.047	− .088	− .001
D_1_ × WMC	0.028	0.024	− 1.16	.247	− .020	.076
D_2_ × WMC	− 0.002	0.028	− 0.07	.945	− .060	.053
IES						
WMC	− 203.60	33.970	− 5.99	< .001	− 270.858	− 136.335
D_1_	326.01	58.280	5.59	< .001	210.635	441.376
D_2_	209.98	66.11	3.18	0.002	79.104	340.854
D_1_ × WMC	− 107.60	72.19	− 1.49	0.139	− 250.532	35.323
D_2_ × WMC	− 44.11	83.08	− 0.53	0.596	− 208.589	120.360

D_1_ = professional vs. lower levels of expertise; D_2_ = amateur vs. recreational levels of expertise

As in the task without distraction, it is theoretically important to establish whether the lack of interaction really supports the model without interaction or whether data are not sensitive enough to decide between alternative theories. To this end, we computed and compared Bayes factors for models with expertise × WMC interaction and the model without this interaction. Again, we found that the model without interaction is 17.54 as likely as the model with interaction, given the observed data. This is strong evidence against the expertise × WMC interaction.

#### Accuracy rates

The results of moderation analysis on accuracy rates showed that the moderation model accounted for 15.3% of the accuracy under distraction variance, *F*(5, 121) = 4.36, *p* = 0.001. There was a significant effect of the expertise showing more accurate tactical decision-making of professionals (*M* = 0.805, *SD* = 0.093) compared to lower levels of soccer expertise (*M* = 0.743, *SD* = 0.108),* t*(125) =  − 3.47, *p* = 0.001, and more accurate tactical decision-making of amateur (*M* = 0.759, *SD* = 0.103) compared to recreational soccer players (*M* = 0.725, *SD* = 0.112), *t*(125) =—2.01, *p* = 0.047. In addition, higher levels of WMC were associated with more accurate tactical decisions under distraction, *t*(121) = 2.73, *p* = 0.007. There were no significant expertise × WMC interaction across both comparisons: professional vs. lower levels of soccer expertise (D_1_ × WMC), *t*(121) = 1.16, *p* = 0.247, and amateur vs. recreational levels (D_2_ × WMC), *t*(125) = − 0.70, *p* > 0.250 (Table 3, Fig. 3B). Corroborating the RTs analysis, Bayes factors on accuracy rates also revealed that the model without interaction is 7.03 as likely as the model with interaction given the observed data.

#### IES analysis

Analysis on IESs showed that the model accounted for 36.2% of the variance, *F*(5, 121) = 13.71, *p* < 0.001. As in the RT analysis, professional soccer players were faster (*M* = 708 ms, *SD* = 284.69) than amateur and recreational soccer players (*M* = 987 ms, *SD* = 372.70), *t*(125) = 5.59, *p* < 0.001, whereas amateur soccer players (*M* = 917 ms, *SD* = 360.94) made faster decisions than recreational soccer players (*M* = 1,064 ms, *SD* = 374.53), *t*(125) = 3.18, *p* = 0.002.

Higher levels of WMC were associated with faster tactical decisions, *t*(121) = − 5.99, *p* = 0.001. The expertise × WMC interactions were not significant, as shown by the comparison between professional soccer players and players with lower levels of expertise (D_1_ × WMC), *t*(121) =  − 1.49, *p* = 0.139, and by the comparison between amateur and recreational soccer players (D_2_ × WMC), *t*(121) =  − 0.53, *p* > 0.250. The regression slopes are depicted in Fig. 3C. Bayes factors on IESs showed that the model without interaction is 7.76 as likely as the model with interaction, given the observed data. This provides further evidence against expertise × WMC interaction.

#### Own name detection in distracting auditory stimuli

The results of logistic regression indicated the independence of the frequency of own name detection in the auditory distraction stimuli from WMC, as well as the independence from expertise and expertise × WMC interaction (Overall model, *χ*^*2*^(3) = 5.08, *p* = 0.166). In other words, soccer players noticed (61.5%) and did not notice (38.5%) their names equally as often in distracting auditory stimuli while solving the tactical decision-making task, regardless of their WMC and level of expertise.

## Discussion

We developed two new tactical decision-making tasks to examine the relationship between WMC and levels of expertise in predicting the performance of soccer players. In both tasks, we found faster and more accurate decision-making of professional soccer players compared to amateur and recreational players. This is expected and reaffirms the "power" of skills and knowledge gained through deliberate practice. We also found that WMC predicted decision-making speed and accuracy in both tasks. Importantly, and contrary to the circumvention-of-limits hypothesis predictions, we found no interaction between expertise and WMC. This finding suggests that WMC is a unique and equally important predictor of tactical performance at all levels of soccer expertise. This is consistent with the independent influence hypothesis and the assumption that the effect of WMC is not reduced at high levels of expertise when tested in ecologically valid dynamic tasks involving constantly changing input (Hambrick et al., 2012).

Our results provide further empirical support for models that assume central control mechanism such as central executive, executive control, or controlled attention (Baddeley & Logie, 1999; Cowan, 1999; Engle et al., 1999). Central control implies a general (domain-free) limited capacity mechanism that controls and regulates the working memory system in the service of complex cognition. From the LT-WM perspective (Ericsson & Kintsch, 1995), such a general mechanism does not determine expert performance due to extensive knowledge and skill-based retrieval cues that enable them to circumvent basic cognitive processing limitations. It should also be noted that the models that assume a central control mechanism do not deny the contribution of domain-specific knowledge and expertise. However, such models postulate that the central control mechanism is responsible for encoding retrieval structures appropriate for incoming stimuli (for theoretical discussion, see Miyake & Shah, 1999) and may contribute to the performance even at high levels of expertise. The lack of WMC × expertise interaction supports this assumption and suggests that the WMC contribution to the prediction of expert performance goes above and beyond domain-specific knowledge and that the deliberate practice is not always sufficient to overcome WMC limitations (Hambrick & Engle, 2002; Hambrick & Oswald, 2005; Meinz & Hambrick, 2010; Meinz et al., 2012).

Criticizing studies that failed to find a reduction of the effect of WMC at the expert level, Ericsson (2014) noted that operationalizations of deliberate practice are often unclear and too broad in studies that failed to find a reduction of the effect of WMC at the expert level. He argued that not every hour of training is necessarily deliberate practice. However, we made every effort to eliminate this objection by strictly following the conceptualization of deliberate practice offered by Ericsson et al. (1993). Based on their soccer practice and competition, we categorized professional soccer players as experts if they were involved in structured soccer training in clubs or academies for ten or more years and played on a professional level for not less than two seasons. Finally, as Meinz et al. (2012) highlighted for the sample of poker players in their study, we would also like to emphasize that we did not include exceptional soccer players in our sample (e.g., most athletes were not Champions League soccer players). Thus, there is a small possibility that WMC plays a less critical role in tactical decision-making in such an extreme group of participants as elite soccer players.

The study of Furley and Memmert (2012), which is, to our knowledge, the only study that adapted the selective attention paradigm (Conway et al., 2001) to examine decision-making in sports, found that high-WMC basketball players were less likely to detect their own names in distracting auditory stimuli than low-WMC players. This is consistent with the models of working memory that incorporate attention control functions as an essential part of the working memory system (Baddeley & Logie, 1999; Cowan, 1999; Engle et al., 1999), and in particular, with the controlled attention theory of WMC (Engle et al., 1999) which attribute working memory limitations to inhibitory processes. By contrast, we found the independence of the WMC and the frequency of own name detection. To account for discrepant findings between the two studies, it should be noted that attention is a multi-component construct, including automatic bottom-up orienting and voluntary top-down control (for a review, see Fougnie 2008). Thus, the appearance of the own name in our dynamic task probably tapped into bottom-up orienting of attention unrelated to WMC, as some studies showed (e.g. Redick & Engle, 2006). More specifically, the appearance of own name in Furley and Memmert's task with a rapid succession of photo stills of tactical situations engaged voluntary control of attention to a greater degree, resulting in a lower rate of name detection of high-WMC athletes. On the other hand, video sequences of tactical situations in our task arguably enabled athletes to form more complex representations of tactical situations with more attentional resources left available to bottom-up orienting stimuli, such as their own name in the distraction stimuli.

## Conclusion

It is not surprising to observe the superiority of professional soccer players in tactical decision-making. An important factor contributing to this superiority is the amount of time experts spend sharpening relevant skills, and as Hambrick and Burgoyne (2019, p. 1) recently stated: "no credible scientist believes that expert performance can be explained without recourse to nurture". However, our results support the view that WMC has a unique role in performance across all levels of expertise. This is consistent with the "new look" perspective on expertise (Hambrick et al., 2016), suggesting that WMC is an overlooked piece of the expertise puzzle.

## Supplementary Information

**Additional file 1**. **Table S1.** The relationship between WMC and tactical decision-making across three levels of soccer expertise.

## Data Availability

All data have been made publicly available via the Open Science Framework (OSF) and can be accessed at: https://osf.io/ybdca/files/osfstorage

## Footnotes

1 No statistically significant differences were found in the frequency of watching soccer games and playing soccer videogames between professional, amateur and recreational soccer players.

2 The features of video clips (overall duration, and the duration of last frame) were set based on the results of two pilot studies.

3 The mean is calculated as the weighted average of decision-making RT (or accuracy rates) of amateur and recreational players.
