# Supplementary material for: The role of working memory capacity in soccer tactical decision making at different levels of expertise
Source: Cogn Res Princ Implic. 2023 Mar 29;8:20. doi: 10.1186/s41235-023-00473-2 (PMC10050259; doi:10.1186/s41235-023-00473-2)
Supplement: Supplementary file 1 — Additional file 1. Table S1. The relationship between WMC and tactical decision-making across three levels of soccer expertise. [file 41235_2023_473_MOESM1_ESM.docx]

**Supplemental material**

**Table S1**

*The relationship between WMC and tactical decision-making across three levels of soccer expertise*

|  | Tactical decision making | | | Tactical decision making under distraction | | |
| --- | --- | --- | --- | --- | --- | --- |
|  | RT | ACC | IES | RT | ACC | IES |
| Professional | -.18 | .14 | -.20 | -.34* | .11 | -.37* |
| Amateur | -.35* | .05 | -.36* | -.42** | .30* | -.44** |
| Recreational | -.38* | .02 | -.32* | -.42* | .30* | -.60** |
| Note. **p* < .05; ***p* < .01 RT = Response time; ACC = Accuracy rates; IES = Inverse efficiency score | | | | | | |
